# In search of a window of opportunity in axial spondyloarthritis: treatment outcomes across different symptom durations

**DOI:** 10.1016/j.ero.2026.03.021

**Published:** 2026-04-23

**Authors:** Annik Steimer, Andrea Götschi, Sofia Ramiro, Michael J. Nissen, Kristina Bürki, Michael Andor, Simon Grosswiler, Diego Kyburz, Burkhard Möller, Sabine Adler, Diana Dan, Laure Brülhart Bletsas, Frauke Förger, Oliver Distler, Raphael Micheroli, Adrian Ciurea

**Affiliations:** 1 Department of Rheumatology, Zurich University Hospital, University of Zurich, Zurich, Switzerland; 2 Swiss Clinical Quality Management in Rheumatic Diseases Foundation, Zurich, Switzerland; 3 Department of Rheumatology, Leiden University Medical Center, Leiden, Netherlands; 4 Department of Rheumatology, Zuyderland Medical Centre Heerlen, Heerlen, Netherlands; 5 Department of Rheumatology, Geneva University Hospital, Geneva, Switzerland; 6 Rheumatologie im Zürcher Oberland, Uster, Switzerland; 7 Ankylosing Spondylitis Association, Zurich, Switzerland; 8 Department of Rheumatology, University Hospital Basel, University of Basel, Basel, Switzerland; 9 Department of Rheumatology and Immunology, Inselspital, University Hospital Bern, Bern, Switzerland; 10 Department of Rheumatology, Cantonal Hospital Aarau, Aarau, Switzerland; 11 Department of Rheumatology, Lausanne University Hospital, University of Lausanne, Lausanne, Switzerland; 12 Division of Rheumatology, La Chaux-de-Fonds Hospital, La Chaux-de-Fonds, Switzerland; 13 Department of Rheumatology, Cantonal Hospital St. Gallen, St. Gallen, Switzerland

## Abstract

**Objectives:**

This study aimed to determine the effectiveness of a first tumour necrosis factor inhibitor (TNFi) in patients with short and long symptom duration in axial spondyloarthritis (axSpA), using alternative cut-offs for back pain duration: ≤1 year as very early, >1 year, and ≤5 years as intermediate axSpA, and >5 years as late axSpA.

**Methods:**

A total of 1161 patients with axSpA from the Swiss Clinical Quality Management registry, who had data on the duration of back pain and initiated their first TNFi, were included. Patients were categorised as follows: very early axSpA (*N* = 138), intermediate axSpA (*N* = 329), and late axSpA (*N* = 694). Adjusted logistic regression models were used to compare the likelihood of achieving a low disease activity status (Axial Spondyloarthritis Disease Activity Score [ASDAS] <2.1) at 1 year, adjusting for age, sex, human leucocyte antigen-B27, educational level, body mass index, ASDAS, current smoking, and presence of inflammation on magnetic resonance imaging of the sacroiliac joints. Drug survival was analysed using multiple-adjusted Cox proportional hazards models. Missing data were addressed through multiple imputation by chained equations.

**Results:**

Higher disease activity parameters were found at baseline in very early axSpA. However, no significant differences were observed in treatment response or TNFi retention between very early and late axSpA (odds ratio for ASDAS < 2.1 response 0.99, 95% CI: 0.62-1.59 and hazard ratio for drug discontinuation 1.09, 95% CI: 0.87-1.37).

**Conclusions:**

Based on this real-world observational data, no evidence was found to suggest a superior effectiveness of TNFi in patients with very early axSpA compared with those with late axSpA.

**WHAT IS ALREADY KNOWN ON THIS TOPIC**

• The Assessment in SpondyloArthritis international Society has defined early axial spondyloarthritis (axSpA) as having ≤2 years of axial symptom duration.
• Start of a biological or targeted-synthetic disease-modifying antirheumatic drug during this early phase of axSpA has not been associated with improved clinical short-term outcomes.

**WHAT THIS STUDY ADDS**

• Initiating tumour necrosis factor inhibitor (TNFi) within 1 year of back pain onset in axSpA does not appear to be associated with better treatment effectiveness compared with starting treatment after 5 years of symptom onset.

**HOW THIS STUDY MIGHT AFFECT RESEARCH, PRACTICE OR POLICY**

• Although early TNFi treatment in axSpA may not provide short-term clinical benefits, its potential to prevent long-term spinal structural progression justifies further exploration in future studies.

Alt-text: Unlabelled box dummy alt text

## INTRODUCTION

Limited evidence exists supporting better outcomes in axial spondyloarthritis (axSpA) if patients are treated early [1,2]. The Assessment of SpondyloArthritis international Society (ASAS) has recently published a consensus definition of early axSpA, intended for research settings, which is characterised by ≤2 years of axial symptoms [3]. However, when this 2-year cut-off was applied to differentiate between early and established axSpA in cohort studies or *post hoc* analyses of randomised-controlled trials assessing biological or targeted-synthetic disease-modifying antirheumatic drugs (b/tsDMARDs), patients with early axSpA did not demonstrate better short-term outcomes [[4], [5], [6]]. A recent meta-analysis of 11 earlier bDMARD trials, using various cut-off points ranging from 2 to 5 years of symptom duration, aimed to reassess the issue of potential better treatment efficacy in early disease [7]. Unlike previous analyses, this meta-analysis accounted for differences in baseline disease characteristics and placebo responses when comparing treatment outcomes between early and established axSpA, ultimately finding no significant benefits of early treatment across all symptom duration cut-offs [7].

Even when the threshold for early disease was lowered to 1 year of symptom duration in an observational real-life setting, treatment with tumour necrosis factor inhibitors (TNFi) did not demonstrate greater effectiveness at this very early stage when compared with established axSpA (symptoms >2 years) [8]. We hypothesised that the difference in symptom durations between <1 year and >2 years might have been too narrow to reveal meaningful differences in treatment response. To address this limitation, we sought to increase the contrast in symptom duration to enhance the ability to detect potential differences in treatment effectiveness.

In this study, we therefore investigated whether improved treatment effectiveness could be observed in very early axSpA (symptoms ≤1 year) when compared with intermediate (>1 and ≤5 years) and late axSpA (>5 years).

## METHODS

### Study population

We included patients with a diagnosis of axSpA recruited by their treating rheumatologist in the Swiss Clinical Quality Management in Rheumatic Diseases (SCQM) registry if the date of onset of back pain was known and if they initiated treatment with a first TNFi [4]. As an alternative to the current definition of early vs established axSpA (≤2 years vs >2years of axial symptom duration), we defined 3 disease stages at start of treatment based solely on back pain duration, as information on morning stiffness was not available: (i) *very early axSpA* (≤1 year); (ii) *intermediate axSpA* (>1 year and ≤5 years); and (iii) *late axSpA* (>5 years). Approval from the Ethics Committee of the Canton of Zurich was obtained (BASEC 2022-00272).

### Treatment effectiveness

Effectiveness of treatment with a first TNFi was investigated with treatment response analyses: achievement of the following outcomes at 1 year (±6 months) after treatment initiation: low disease activity status (Axial Spondyloarthritis Disease Activity Score [ASDAS] < 2.1), inactive disease status (ASDAS < 1.3), clinically important improvement in ASDAS (ASDAS-CII), and major improvement in ASDAS (ASDAS-MI). If patients discontinued treatment before response assessment for other reasons than remission, they were considered nonresponders. Drug retention was analysed as a secondary outcome.

### Statistical analyses

The following statistical tests were used to compare baseline characteristics at the start of treatment between patients with very early, intermediate, and late axSpA: the Kruskal-Wallis Rank Sum test for continuous variables and the Chi-square test for categorical variables (significance level set to 0.05). We used logistic regression analyses to estimate adjusted odds ratios (ORs) for treatment response in patients with very early vs late disease and in patients with intermediate disease vs late disease. The models were adjusted for the following confounders: age, sex, human leucocyte antigen-B27 (HLA-B27) status, educational level, ASDAS, inflammatory sacroiliac magnetic resonance imaging (MRI) changes, body mass index (BMI), and smoking status. We used multiple imputation by chained equations (MICE) to account for missing data (see below) [9] and performed complete case analyses as sensitivity analyses. The same covariates were included in multiple-adjusted Cox proportional hazards models to estimate hazard ratios (HRs) for drug discontinuation in patients with very early axSpA vs late axSpA and in patients with intermediate axSpA vs late axSpA.

For MICE, we generated 65 imputations using predictive mean matching for continuous variables, logistic regression for binary variables, and proportional-odds models for ordered variables [9]. All covariates and all components of the outcomes were included in the imputation models for the response analyses [10]. For the retention analysis, covariates were imputed based on all covariates, the time-to-event variable, and the event indicator. We used trace plots and compared the distribution of observed and imputed values to assess the quality of the multiple imputation. Estimates were pooled using Rubin’s rules [11]. The MICE package version 3.17.0 was used [9].

## RESULTS

A total of 1161 patients with axSpA fulfilled the inclusion criteria (very early axSpA [*N* = 138], intermediate axSpA [*N* = 329], and late axSpA [*N* = 694]). Baseline characteristics at the start of treatment with the first TNFi are shown in Table 1. Patients with very early axSpA had a higher prevalence of objective signs of inflammation, including elevated C-reactive protein levels and sacroiliitis on MRI, whereas patients with late axSpA were more likely to be HLA-B27 positive. There was a trend towards higher Bath Ankylosing Spondylitis Disease Activity Index scores in very early axSpA. Although patients with late axSpA had a higher impairment in spinal mobility, global functional impairment was similar across the groups. The prevalence of peripheral musculoskeletal manifestations was comparable in all 3 groups.

**Table 1:** Characteristics of patients with axSpA with very early disease, intermediate disease, and late disease at the start of the first TNF inhibitor

			Duration of back pain				
Parameter	N	Very early axSpA (≤1 y) N = 138	N	Intermediate axSpA (>1 and ≤5 y) N = 329	N	Late axSpA (>5 y) N = 694	*P*
Male sex, N (%)	138	76 (55)	329	156 (47)	694	371 (54)	.14
Age (y)	138	37.9 (13.1)	329	38.6 (12.6)	694	45.3 (11.7)	<.001
Symptom duration, y, median (IQR)	138	0.9 (0.6-5.6)	323	3.0 (2.0-4.3)	690	14.3 (9.0-22.9)	<.001
Back pain duration, y, median (IQR)	138	0.5 (0.3-0.7)	329	2.5 (1.7-3.6)	694	14.3 (8.9-22.5)	<.001
HLA-B27, N (%)	123	71 (58)	302	172 (57)	637	440 (69)	<.001
Body mass index	127	24.5 (4.2)	293	25.5 (4.5)	640	25.8 (4.7)	.01
Education	114		258		571		.06
• Compulsory, N (%)		17 (15)		42 (16)		92 (16)	
• Vocational, N (%)		75 (66)		157 (61)		309 (54)	
• Academic, N (%)		22 (19)		59 (23)		170 (30)	
ASAS classification criteria fulfilled, N (%)	116	87 (75)	252	198 (79)	531	475 (90)	<.001
IBP (ASAS definition fulfilled) (ever), N (%)	122	79 (65)	260	175 (67)	536	453 (85)	<.001
Sacroiliitis on MRI present (inflammation), N (%)	130	75 (58)	284	172 (61)	598	242 (41)	<.001
Definite sacroiliitis on radiograph present, N (%)	130	27 (21)	288	46 (16)	604	84 (65)	<.001
Peripheral arthritis (ever), N (%)	134	65 (49)	304	125 (41)	639	277 (43)	.36
Enthesitis (ever), N (%)	134	91 (68)	299	205 (69)	632	440 (70)	.90
Uveitis (ever), N (%)	103	6 (6)	232	17 (7)	498	94 (19)	<.001
Psoriasis (ever), N (%)	103	12 (12)	240	25 (10)	505	59 (12)	.87
Inflammatory bowel disease (ever), N (%)	101	8 (8)	231	10 (4)	493	49 (10)	.04
Physician global disease activity	123	5.3 (1.9)	269	4.7 (1.8)	571	4.7 (1.9)	.01
Patient global disease activity	106	6.4 (2.3)	226	6.0 (2.3)	514	6.0 (2.5)	.37
BASDAI	105	5.7 (1.9)	212	5.3 (2.0)	503	5.3 (2.0)	.09
ASDAS	96	3.4 (0.9)	209	3.1 (0.9)	485	3.2 (0.9)	.01
Elevated CRP, N (%)	119	60 (50)	267	111 (42)	543	252 (46)	.22
CRP (mg/L), median (IQR)	120	6.8 (3.0-16.0)	267	5.0 (2.0-11.0)	544	6.3 (2.0-14.0)	.03
BASFI	104	3.9 (2.4)	209	3.4 (2.3)	485	3.7 (2.4)	.26
BASMI	113	1.5 (1.4)	241	1.3 (1.3)	510	2.3 (2.0)	<.001

ASAS, Assessment of SpondyloArthritis international Society; ASDAS, Axial Spondyloarthritis Disease Activity Score; axSpA, axial spondyloarthritis; BASDAI, Bath Ankylosing Spondylitis Disease Activity Index; BASFI, Bath Ankylosing Spondylitis Functional Index; BASMI, Bath Ankylosing Spondylitis Metrology Index; CRP, C-reactive protein; HLA-B27, human leucocyte antigen-B27; IBP, inflammatory back pain; MRI, magnetic resonance imaging; TNF, tumour necrosis factor.

Presented statistics are means (SD), except where otherwise indicated.

### TNFi response analyses

The results for all response outcomes at 1 year, after adjustment for potential confounders and multiple imputation of missing covariates, are presented in Table 2. No difference in response rates was observed between very early and late axSpA: OR 0.99 (95% CI: 0.62-1.59) for ASDAS < 2.1, OR 0.83 (95% CI: 0.46-1.49) for ASDAS < 1.3, OR 0.89 (95% CI: 0.53-1.49) for ASDAS-CII, and OR 0.82 (95% CI: 0.41-1.61) for ASDAS-MI (Table 2). Likewise, no significant differences in response rates were observed for any of the analysed outcomes between intermediate and late axSpA (Table 2). Comparable results were found in the complete case models (Table 3).

**Table 2:** Multiple-adjusted response rate analyses at 1 y of treatment with a first TNFi for different outcomes in very early and intermediate vs late axSpA after adjustment for potential confounders (N = 1161)

	ASDAS < 2.1			ASDAS < 1.3			ASDAS-CII			ASDAS-MI		
Variable	OR	95% CI	*P*	OR	95% CI	*P*	OR	95% CI	*P*	OR	95% CI	*P*
Very early vs late axSpA	0.99	0.62-1.59	.97	0.83	0.46-1.49	.53	0.89	0.53-1.49	.65	0.82	0.41-1.61	.55
Intermediate vs late axSpA	0.88	0.62-1.26	.49	0.69	0.43-1.12	.13	0.79	0.53-1.17	.24	0.82	0.47-1.41	.46
Age	0.98	0.97-1.00	.01	0.98	0.96-1.00	.02	0.99	0.97-1.00	.04	0.99	0.97-1.00	.14
Female sex	0.42	0.31-0.56	<.001	0.37	0.24-0.55	<.001	0.43	0.31-0.59	<.001	0.36	0.23-0.58	<.001
HLA-B27 negativity	0.40	0.28-0.56	<.001	0.41	0.25-0.67	<.001	0.39	0.27-0.57	<.001	0.38	0.22-0.67	<.001
Education vocational vs compulsory	1.66	1.01-2.74	.047	1.78	0.85-3.70	.12	1.62	0.99-2.67	.056	1.53	0.78-3.03	.22
Education academic vs compulsory	2.43	1.42-4.15	.001	2.13	0.97-4.67	.06	2.20	1.25-3.86	.01	2.33	1.11-4.86	.03
Body mass index	0.96	0.93-1.00	.04	0.92	0.87-0.97	.001	0.97	0.93-1.00	.08	0.93	0.88-0.98	.01
Current smoking	0.89	0.65-1.22	.48	0.68	0.45-1.02	.06	0.88	0.63-1.24	.47	0.82	0.52-1.29	.40
ASDAS	0.75	0.63-0.89	.001	0.78	0.63-0.98	.03	2.80	2.27-3.46	<.001	5.91	4.30-8.13	<.001
Sacroiliitis on MRI (inflammation)	1.17	0.85-1.61	.35	1.27	0.86-1.86	.23	1.09	0.78-1.50	.62	1.23	0.76-1.99	.39

ASDAS, Axial Spondyloarthritis Disease Activity Score; ASDAS-CII, clinically important improvement in ASDAS; ASDAS-MI, major improvement in ASDAS; axSpA, axial spondyloarthritis; HLA-B27, human leucocyte antigen-B27; MRI, magnetic resonance imaging; OR, odds ratio; TNFi, tumour necrosis factor inhibitor..

Response rates in patients with available outcome at 1 y (±6 mo), patients who had discontinued the biologic in the meantime for reasons other than remission were considered nonresponders.

**Table 3:** Multiple-adjusted response rate analyses at 1 y of treatment with a first TNFi for different outcomes in very early and intermediate vs late axSpA after adjustment for potential confounders (complete case analysis)

	ASDAS < 2.1			ASDAS < 1.3			ASDAS-CII			ASDAS-MI		
Variable	OR	95% CI	*P*	OR	95% CI	*P*	OR	95% CI	*P*	OR	95% CI	*P*
Very early vs late axSpA	1.51	0.75-3.06	.25	0.60	0.24-1.39	.25	1.17	0.57-2.43	.67	0.97	0.37-2.42	.95
Intermediate vs late axSpA	0.93	0.53-1.62	.80	0.56	0.27-1.10	.10	0.77	0.43-1.36	.37	1.11	0.50-2.42	.80
Age	0.99	0.97-1.01	.29	0.98	0.95-1.00	.06	0.99	0.97-1.01	.58	1.00	0.97-1.02	.83
Female sex	0.32	0.20-0.51	<.001	0.33	0.18-0.60	<.001	0.37	0.22-0.59	<.001	0.27	0.13-0.54	<.001
HLA-B27 negativity	0.44	0.26-0.72	.002	0.40	0.19-0.79	<.001	0.46	0.27-0.78	.004	0.44	0.19-0.96	.04
Education vocational vs compulsory	2.34	1.15-4.96	.02	3.05	1.15-9.75	.04	2.06	1.03-4.27	.046	1.33	0.53-3.57	.55
Education academic vs compulsory	3.22	1.50-7.17	.003	2.82	1.01-9.35	.06	2.68	1.26-5.91	.01	2.00	0.73-5.80	.19
Body mass index	0.94	0.89-1.00	.04	0.89	0.81-0.96	.004	0.94	0.88-0.99	.03	0.86	0.78-0.94	.002
Current smoking	0.91	0.56-1.48	.71	0.61	0.33-1.08	.10	0.84	0.51-1.37	.48	0.60	0.30-1.16	.13
ASDAS	0.65	0.49-0.84	.001	0.63	0.46-0.85	.003	2.85	2.12-3.91	<.001	6.53	4.13-11.0	<.001
Sacroiliitis on MRI (inflammation)	1.40	0.88-2.22	.15	1.83	1.06-3.17	.03	1.21	0.76-1.96	.42	1.56	0.81-3.03	.19
(Number patients/number events)	(396/176)			(396/85)			(396/166)			(396/75)		

ASAS, Assessment of SpondyloArthritis international Society; ASDAS, Axial Spondyloarthritis Disease Activity Score; ASDAS-CII, clinically important improvement in ASDAS; ASDAS-MI, major improvement in ASDAS; axSpA, axial spondyloarthritis; HLA-B27, human leucocyte antigen-B27; MRI, magnetic resonance imaging; OR, odds ratio; TNFi, tumour necrosis factor inhibitor.

Response rates in patients with available outcome at 1 y (±6 mo), patients who had discontinued the biologic in the meantime for reasons other than remission being considered nonresponders.

### TNFi retention analyses

We observed a difference in median unadjusted survival of the first TNFi: 2.3 years (95% CI: 1.5-2.9) in very early axSpA, 1.8 years (95% CI: 1.2-2.6) in intermediate axSpA, and 2.6 years (95% CI: 2.2-3.1) in late axSpA (*P* value Log-rank test .02). The reasons for drug discontinuation were similarly distributed in the 3 groups (Table 4).

**Table 4:** Reason for discontinuation of the first TNFi

		Duration of back pain			
Parameter	N	Very early axSpA (≤1 y) N = 96	Intermediate axSpA (>1 and ≤5 y) N = 229	Late axSpA (>5 y) N = 445	Overall *P* value
	770				.54
Adverse events, N (%)		9 (9.4)	28 (12.2)	75 (16.8)	
Inefficacy, N (%)		35 (36.5)	94 (41.0)	177 (39.8)	
Preference, N (%)		0	0	3 (0.7)	
Pregnancy, N (%)		0	1 (0.4)	0	
Remission, N (%)		4 (4.2)	14 (6.1)	15 (3.4)	
Other, N (%)		18 (18.8)	34 (14.8)	65 (14.6)	
Reason not provided, N (%)		30 (31.2)	56 (24.5)	110 (24.7)	

axSpA, axial spondyloarthritis; TNFi, tumour necrosis factor inhibitor.

After adjusting for age, sex, HLA-B27, educational level, ASDAS, sacroiliac joint inflammation on MRI, BMI, and current smoking, the estimated hazards of discontinuing the first TNFi were comparable between the groups: HR 1.09 (95% CI: 0.87-1.37) for very early vs late axSpA and HR 1.16 (95% CI: 0.98-1.37) for intermediate vs late axSpA (Table 5). Comparable results were found in the complete case analysis (Table 6).

**Table 5:** Multiple-adjusted Cox proportional hazards model for analysis of drug discontinuation of a first TNF inhibitor in very early and intermediate vs late axial spondyloarthritis (N = 1161)

Variable	HR	95% CI	*P*
Very early vs late axSpA	1.09	0.87-1.37	.46
Intermediate vs late axSpA	1.16	0.98-1.37	.09
Age	0.99	0.99-1.00	.59
Female sex	1.58	1.35-1.84	<.001
HLA-B27 negativity	1.45	1.23-1.71	<.001
Education vocational vs compulsory	0.97	0.77-1.22	.78
Education academic vs compulsory	0.88	0.68-1.14	.34
Body mass index	1.01	0.99-1.03	.24
Current smoking	1.14	0.97-1.35	.12
ASDAS	0.92	0.84-1.01	.07
Sacroiliitis on MRI (inflammation)	0.95	0.81-1.11	.48

ASDAS, Axial Spondyloarthritis Disease Activity Score; axSpA, axial spondyloarthritis; HLA-B27, human leucocyte antigen-B27; MRI, magnetic resonance imaging; TNF, tumour necrosis factor.

**Table 6:** Multiple-adjusted Cox proportional hazards model for analysis of drug discontinuation of a first TNF inhibitor in very early and intermediate vs late axial spondyloarthritis (complete case analysis; N = 590)

Variable	HR	95% CI	*P*
Very early vs late axSpA	0.82	0.60-1.12	.22
Intermediate vs late axSpA	1.04	0.82-1.32	.73
Age	0.99	0.98-1.00	.10
Female sex	1.65	1.33-2.04	<.001
HLA-B27 negativity	1.35	1.09-1.68	.01
Education vocational vs compulsory	0.94	0.70-1.25	.65
Education academic vs compulsory	0.99	0.72-1.36	.96
Body mass index	1.02	0.99-1.04	.15
Current smoking	1.13	0.91-1.40	.28
ASDAS	0.87	0.78-0.98	.02
Sacroiliitis on MRI (inflammation)	0.84	0.69-1.02	.08

ASDAS, Axial Spondyloarthritis Disease Activity Score; axSpA, axial spondyloarthritis; HLA-B27, human leucocyte antigen-B27; HR, hazard ratio; MRI, magnetic resonance imaging; TNF, tumour necrosis factor.

## DISCUSSION

This study, performed within the real-world setting of a clinical registry, contributes to the growing body of evidence that the short-term effectiveness of bDMARDs in axSpA is not affected by the duration of symptoms [[3], [4], [5], [6], [7], [8]]. Although drug survival varied across very early, intermediate, and late axSpA stages, the difference disappeared after adjusting for potential confounders, suggesting that baseline characteristics—rather than symptom duration—accounted for the observed effect. Our findings suggest that initiating treatment at any point in the disease course yields similar short-term outcomes. Patients can therefore expect to achieve a comparable level of clinical improvement regardless of when therapy is started. Considering that MRI changes—particularly bone marrow oedema—in early disease are often nonspecific and may increase the risk of overdiagnosis [12], it may be reasonable to delay the initiation of immunosuppressive treatment if the diagnosis remains uncertain. The ASAS recommends reassessing the diagnosis before switching bDMARDs in axSpA when the first bDMARD shows insufficient efficacy [13]. In this context, delaying the start of the first bDMARD until the diagnosis is confirmed could be a prudent alternative.

The potential for long-term clinical benefits—both in terms of treatment effectiveness and slowing down radiographic progression, which together affect quality of life [14,15]—remains uncertain, and has not yet been thoroughly investigated. It is important to note that the current data do not rule out the existence of a ‘window of opportunity’ in axSpA [16]. This concept, originally introduced in rheumatoid arthritis (RA), refers to a critical period during which early initiation of treatment can inhibit the evolution of structural articular damage and, consequently, long-term functional impairment [17]. Unlike RA, where structural damage is primarily driven by erosive articular changes, axSpA is characterised by new bone formation at entheseal sites [18,19]. In the spine, this process leads to the formation of bridging syndesmophytes, which are thought to result from excessive reparative bone remodelling [19]. TNFi have been shown to retard spinal radiographic progression in several observational studies [[20], [21], [22], [23], [24]], mainly through inhibition of inflammation. It is hypothesised that minimal new bone formation may occur if inflammation is effectively suppressed early and sustained [19]. However, a recent study comparing radiographic progression in early vs established axSpA found no significant differences [25]. In this study, stratification into early and established axSpA was not performed at diagnosis, but rather at the first available radiograph, limiting the ability to address the question of disease modification through early diagnosis and treatment.

Limitations of the present analysis include its observational design, the restriction to tumour necrosis factor (TNF) inhibitors as the therapeutic modality—reflecting their predominant use as first-line b/tsDMARDs—and the reliance on local MRI readings, as MRI scans are not routinely collected within the SCQM.

In conclusion, although early TNFi treatment in axSpA does not seem to provide short-term clinical benefits, its potential to improve long-term effectiveness, preserve quality of life, and inhibit spinal structural progression—if initiated promptly—warrants further investigation in future studies.

## Editor disclosure

The peer review process did not involve Editorial Board Members Sofia Ramiro and Oliver Distler, and the editorial decision-making was led by editors who were not involved in the creation of this manuscript.

## Competing interests

AS is supported by the MLR Foundation. SR received research grants and/or consulting fees from AbbVie, Alfasigma, Eli Lilly, Johnson & Johnson, MSD, Novartis, Pfizer, Takeda, and UCB. DK received a research grant from AbbVie, honoraria for presentations from AbbVie and Eli Lilly, support for attending meetings from Janssen and Eli Lilly, as well as payments for participation on advisory boards from AbbVie, Eli Lilly, Janssen, Novartis, Pfizer, and Roche. MJN received consulting and/or speaking fees from AbbVie, Eli Lilly, Janssen, Novartis, and Pfizer, as well as a research grant from Novartis. SA received consulting fees and/or speaking fees and/or support for attending meetings from AbbVie, Eli Lilly, Janssen, GSK, Novartis, Pfizer, and Roche. DD received speaking fees and/or support for attending meetings from Eli Lilly, Novartis, UCB, GSK, Menarini, Viatris, AbbVie, AstraZeneca, and Janssen. FF received research grants from Novartis, GSK, and UCB; consulting fees from UCB, GSK, Roche, and Otsuka, and speaking fees from Mepha, UCB, and MSD. OD has consultancy relationships with and/or has received research funding from and/or has served as a speaker for the following companies in the last 3 calendar years: 4P-Pharma, AbbVie, Acepodia, Aera, AnaMar, Anaveon, Argenx, AstraZeneca, Avalyn, Boehringer Ingelheim, BMS, Calluna, Cantargia, CSL Behring, EMD Serono, Galderma, Galapagos, Gossamer, Hemetron, Innovaderm, Kali, Kymera, Lilly, Mediar, MSD Merck, Mitsubishi Tanabe, Nkarta, Novartis, Oorja Bio, Orion, Pliant, Prometheus, Quell, Scleroderma Research Foundation, Skyhawk, Tandem, Topadur, UCB. and Umlaut.bio; he has a patent issued ‘mir-29 for the treatment of systemic sclerosis’ (US8247389, EP2331143) and is a cofounder of CITUS AG. RM received honoraria for lectures or presentations from AbbVie, Eli Lilly, Janssen, Gilead, and Pfizer. AC, AG, BM, KB, LBB, MA, and SG declare they have no conflicts of interest.
